# Computed tomographic findings and treatment of a bull with pituitary gland abscess

**DOI:** 10.1186/s13028-017-0276-1

**Published:** 2017-01-13

**Authors:** Ueli Braun, Alexandra Malbon, Manon Kochan, Barbara Riond, Fredi Janett, Cornelia Iten, Matthias Dennler

**Affiliations:** 1 Department of Farm Animals, Vetsuisse Faculty, University of Zurich, Zurich, Switzerland; 2 Institute of Veterinary Pathology, Vetsuisse Faculty, University of Zurich, Zurich, Switzerland; 3 Clinic of Diagnostic Imaging, Vetsuisse Faculty, University of Zurich, Zurich, Switzerland; 4 Swissgenetics, Mülligen, Switzerland

**Keywords:** Bovine, Brain, Pituitary, Abscess, Computed tomography, Therapy

## Abstract

**Background:**

In cattle, the prognosis of brain abscess is unfavourable and treatment is therefore not recommended. To the knowledge of the authors, there has been no report of successful treatment of a brain abscess in cattle.This report describes the clinical, computed tomographic and postmortem findings in a Holstein–Friesian bull with a hypophyseal abscess.

**Case report:**

The main clinical findings were generalised ataxia, ptyalism, prolapse of the tongue, dropped jaw, dysphagia, head tilt and unilateral ptosis. Cerebrospinal fluid evaluation revealed 2437 leukocytes/µl and severe pleocytosis. CT examination of the head showed a cavitary lesion consistent with an abscess in the hypophysis. Treatment consisted of gentamicin and flunixin meglumine for 3 days and amoxicillin for 40 days. The neurological signs resolved within 8 days of the start of treatment. The bull was slaughtered 11 months later because of infertility, and a postmortem examination was carried out. Histologically, a mild chronic non suppurative meningoencephalitis restricted to the ventral *diencephalon* was diagnosed. In addition, there was mild to moderate multifocal chronic lymphoplasmacytic hypophysitis with mild multifocal fibrosis.

**Conclusions:**

This case report stresses the significance of CT in confirming the clinical and laboratory diagnosis of central nervous system disorders in cattle and for localising brain lesions. Treatment of the brain abscess resulted, with respect to the central nervous disorder, in a successful outcome and was encouraging considering that most cases have an unfavourable prognosis.

## Background

Brain abscesses are relatively rare in cattle, but when present the most common location is in the brainstem [1]; other parts of the brain are only occasionally affected. One of the most common bacterial causes of brain abscess is *Trueperella pyogenes*. In calves, *Fusobacterium necrophorum* may ascend from the oral cavity to the brain, resulting in abscess formation. Other bacteria occasionally isolated include *Actinomyces bovis* and *Mycobacterium bovis* [2]. Bacteria usually gain access to the brain via the bloodstream. Brain infection also may result from oropharyngeal infection with *Listeria monocytogenes*, which ascends the trigeminal nerve in ruminants [2], and from complications associated with dehorning and otitis media [2]. Extension of nasopharyngeal infection after insertion of a nose ring [3] and suppurative encephalitis after a perforating skull fracture [4] also have also been reported. The pituitary region of ruminants is a preferential location for brain abscesses. The pituitary gland is surrounded by the *rete mirabile*, an extensive capillary network, which predisposes the region to bacterial colonisation [2]. The principal clinical signs of pituitary abscess are difficulty prehending and chewing food, dysphagia, excessive salivation, tongue flaccidity and mandibular weakness. Some animals have bradycardia. A tentative diagnosis of brain abscess is based on the clinical signs and may be aided by the results of cerebrospinal fluid (CSF) analysis, although focal suppurative brain lesions are not always accompanied by pleocytosis and increased protein concentration in the CSF. Computed tomography (CT) and magnetic resonance imaging (MRI) have been used successfully for diagnosing brain abscesses in cattle [5, 6]. MRI was used to diagnose a brainstem abscess in a 3-month-old calf [7], and CT was used to image brain abscesses in two calves [6] and a cerebellar abscess in another calf [5].

The prognosis of brain abscess usually is unfavourable [1, 2]. Antibiotics and nonsteroidal anti-inflammatory drugs do not have an appreciable effect on brain abscesses and therefore treatment is usually not recommended. Surgical excision combined with antibiotic treatment is considered the treatment of choice in human patients with pituitary abscess [8–11] but successful treatment with antibiotics alone also has been reported [12]. This case report describes the clinical signs, treatment and postmortem findings in a Holstein–Friesian bull with an abscess in the hypophysis confirmed using CT. The bull was treated successfully, but was slaughtered 11 months later because of poor semen quality.

## Case presentation

A 22-month-old bull was referred to the Department of Farm Animals, University of Zurich, because of pyrexia. Bronchopneumonia caused by *T. pyogenes* was diagnosed based on the results of clinical, radiographic and ultrasonographic examinations and bacterial culture of tracheal secretions. The bull was treated with amoxicillin (7 mg/kg intramuscularly; Clamoxyl^®^, Zoetis Switzerland, Zürich) for 12 days. The bull responded to treatment and was healthy at the time of discharge. The bull was referred again 24 days after the end of the antibiotic treatment because of recurrence of fever in addition to neurological deficits.

At the second referral, the bull was severely obtunded and had low head carriage, poor appetite, decreased rectal temperature (38.2 °C), severe bradycardia [32 beats/min (bpm)] and a normal respiratory rate without any signs of respiratory disease. Neurological examination showed generalised ataxia, hypersalivation, mild prolapse of the tongue, dropped jaw, dysphagia, mild head tilt to the right and ptosis on the right side.

**Fig. 1:**
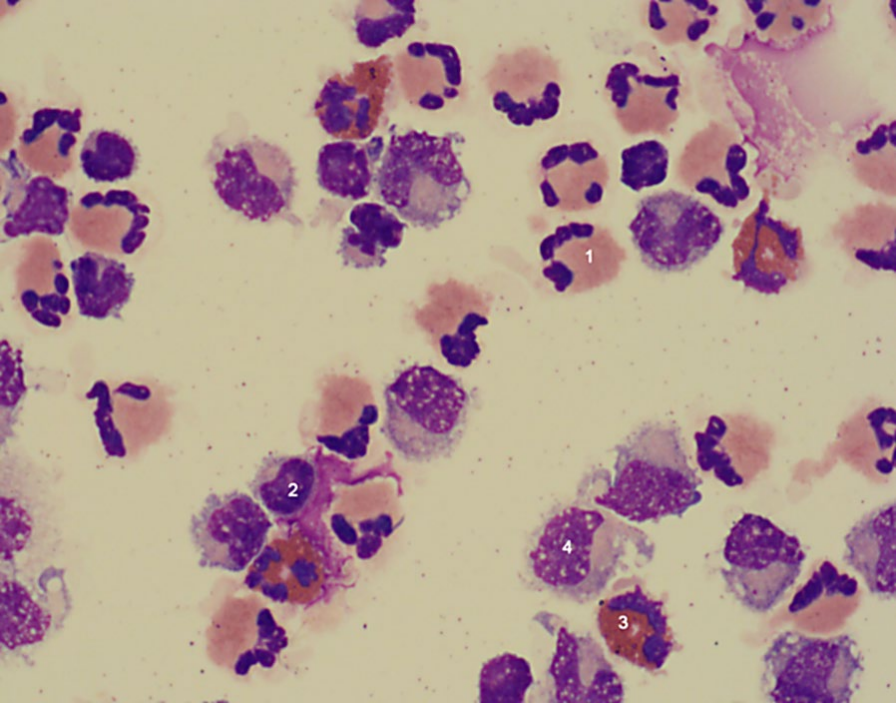
Cerebrospinal fluid. Cytological smear of cerebrospinal fluid from a Holstein–Friesian bull with abscess of the *diencephalon*. There is severe mixed-cell pleocytosis with neutrophils (*1*), lymphocytes (*2*), eosinophils (*3*) and monocytes (*4*)

Haematological analysis showed increased total protein concentration in plasma (90 g/l, reference range 60–80 g/l) and clot formation in the glutaraldehyde test after 4 min (reference range 10–15 min). The pH of a venous blood sample was slightly decreased at 7.37 (reference range 7.40–7.50). All other measured variables (white blood cell count; concentrations of fibrinogen, urea, bilirubin and electrolytes; enzyme activities) were within the reference ranges. CSF collected at the atlanto-occipital foramen under ultrasonographic guidance [13] was colourless, slightly turbid and had a markedly increased leukocyte count of 2437 cells/µl (reference range <10 cells/µl). Cell differentiation showed severe mixed pleocytosis with 59% neutrophils, 26% lymphocytes, 9% eosinophils and 6% monocytes (Fig. 1). The protein concentration in the CSF was markedly increased at 3.1 g/l (reference range <0.75 g/l) [14].

**Fig. 2:**
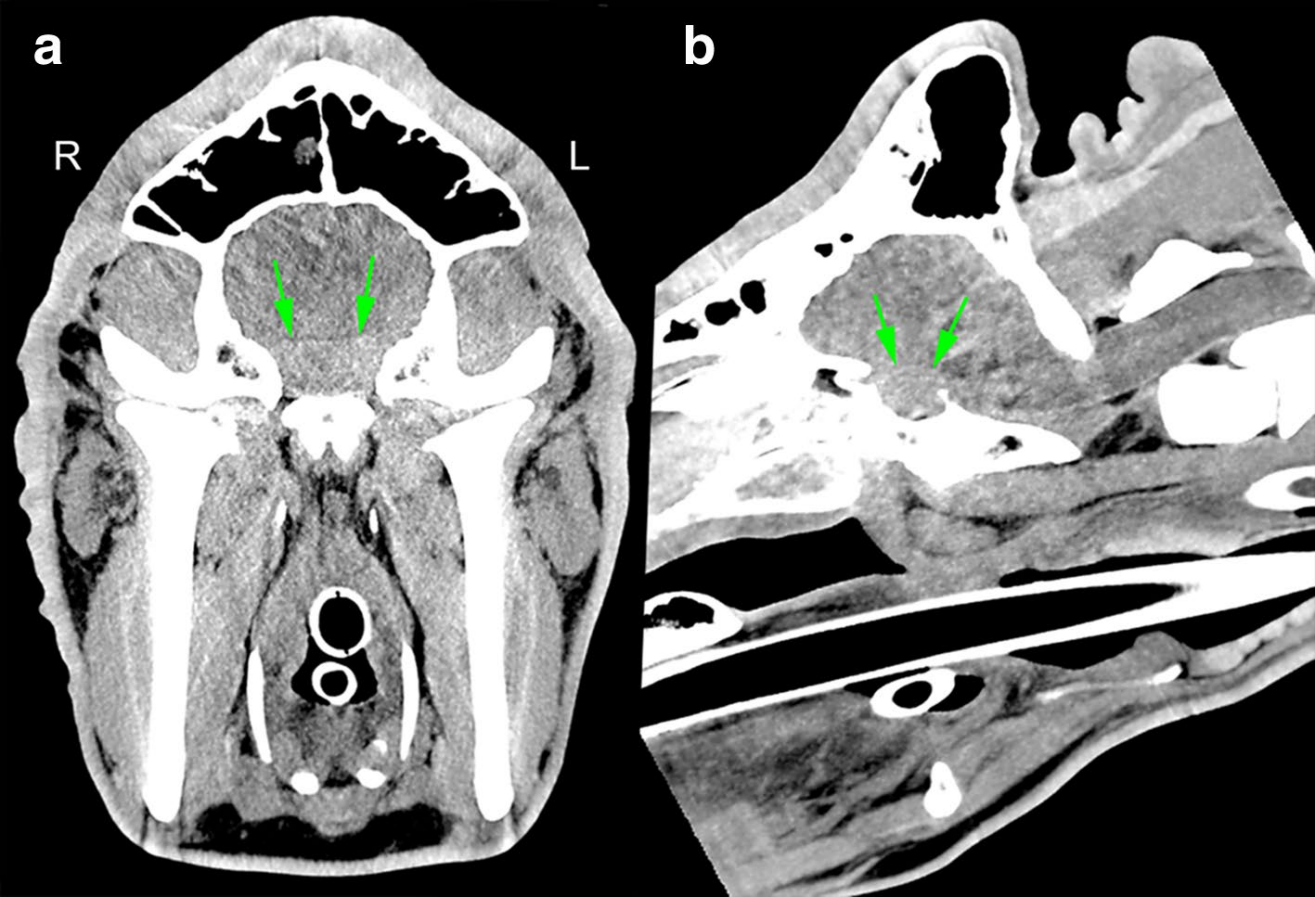
Pre-contrast CT image. Transverse **a** and sagittal **b** pre-contrast CT image reconstruction in a soft tissue algorithm (window level 50, window width 200) of the brain of a Holstein–Friesian bull with abscess of the hypophysis. The hypophysis (*green arrows*) had a heterogeneous attenuation pattern and a higher attenuation value than the surrounding brain parenchyma. The attenuation value was slightly lower on the *left side* of the hypophysis than on the *right side*

**Fig. 3:**
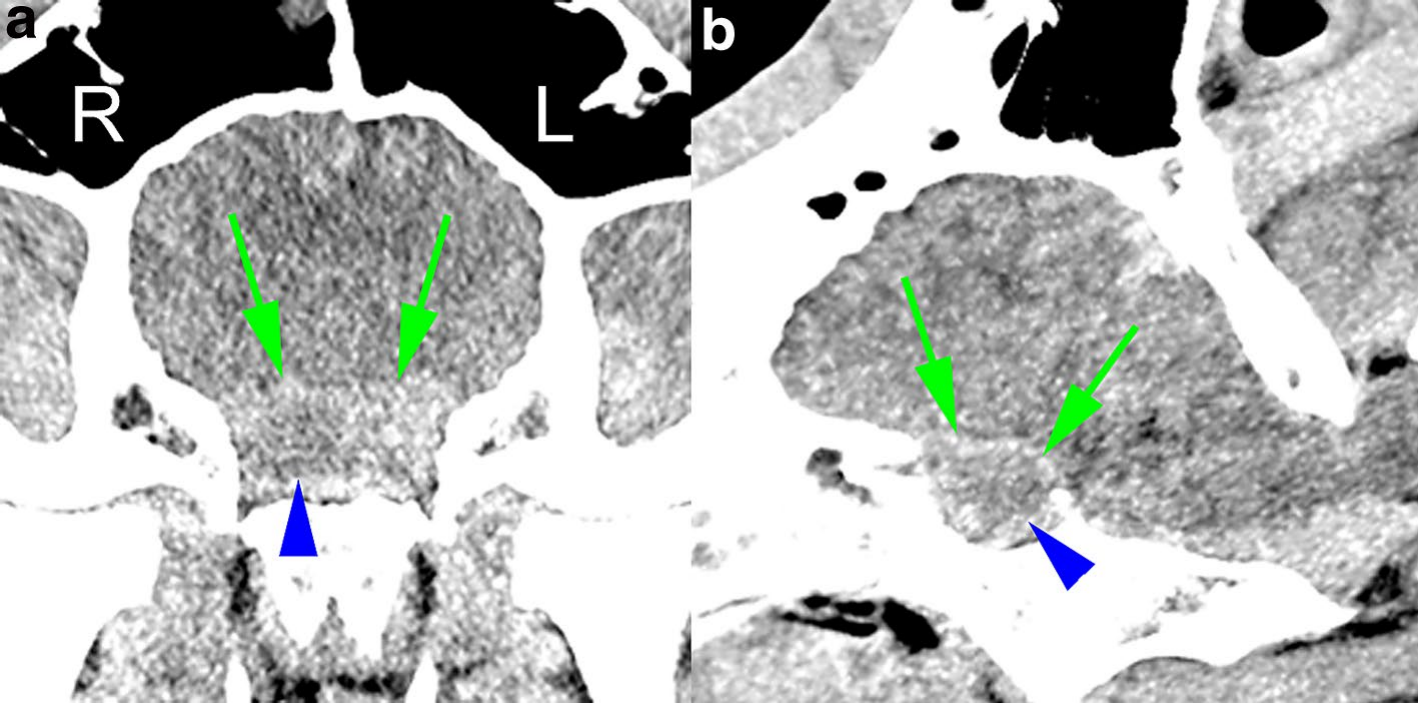
Contrast-enhanced CT image. Transverse **a** and sagittal **b** contrast-enhanced CT image reconstruction in a soft tissue algorithm (window level 50, window width 200) of the brain of a Holstein–Friesian bull with abscess of the hypophysis. The attenuation value of the hypophysis (*green arrows*) was higher than in the pre-contrast image. There is a cavity at the centre of the right side of the hypophysis with low attenuation (*blue arrow heads*). An abscess in the hypophyseal tissue is the most likely diagnosis of this cavitary lesion, whereas a Rathke’s cleft cyst or neoplasm is less likely

A CT examination (Siemens Somatom Sensation Open) was carried out with the bull under general anaesthesia in dorsal recumbency with the following scan parameters: slice thickness 1.5 mm, kVP 140, mA 370, rotation time 1 s, collimator pitch 0.75 with a field of view of 410 × 410 mm. The images were reconstructed in a soft tissue and bone algorithm with a matrix of 512 × 512. The hypophysis had a higher pre-contrast attenuation value than the surrounding brain tissue (Fig. 2) and measured 3.1 × 4.8 × 4.5 cm (height, width, length). The gland occupied the entire *sella turcica*. The attenuation value was slightly lower on the left side of the hypophysis (left: 75.6 ± 17 HU; right: 94.2 ± 16 HU). The CT scan was repeated with the same scan parameters 100 s after intravenous injection (flow rate: 2.5 ml/s) of 600 ml of iohexol, a non-iodic contrast medium (Accupaque 250^®^) and showed that the attenuation value of the right side of the hypophysis was increased to 108.1 ± 19 HU (Fig. 3). There was an irregularly shaped cavern characterised by low attenuation (77 ± 16 HU) at the centre of the right half of the hypophysis. A radiographic diagnosis of a cavitary lesion at the centre of the right side of the hypophysis was made. The most likely aetiology of the lesion was an abscess. Differential diagnoses of the cavitary lesion including a Rathke’s cleft cyst, haematoma or neoplasm were considered less likely because of the asymmetry of the lesion, the pre-contrast attenuation values and the clinical findings.

Based on all the findings, a focal lesion in the right rostrodorsal aspect of the hypophysis, was diagnosed. The results of CSF analysis indicated severe suppurative inflammation and combined with CT findings allowed the diagnosis of an abscess. Even though the prognosis was guarded, the bull underwent treatment because of his high genetic merit. The bull was treated with gentamicin (4 mg/kg; Vetagent^®^, MSD Animal Health, Lucerne), flunixin meglumine (0.75 mg/kg; Fluniximin^®^, Graeub, Berne) administered intravenously for three days, 10 l/day of a solution containing 50 glucose and 9 g NaCl/l administered via an indwelling catheter for four days and amoxicillin (7 mg/kg; Clamoxyl^®^) administered intramuscularly for 40 days. The neurological signs, particularly the dropped lower jaw, protruding tongue and slow jaw movements, remained unchanged for several days but then subsided gradually and resolved by day 9 of treatment. Thereafter, the health status and appetite were normal and the rectal temperature remained in the reference range (38.4–38.9 °C). The heart rate increased to 60 bpm within three days of treatment and was in the reference range (60–66 bpm) on 18 days of the 35-day hospitalisation period. Furthermore, because both testicles decreased in size, testosterone concentration was measured and a semen analysis carried out. The testosterone concentration in serum was decreased at 0.28 ng/ml (reference range 0.4–3.0 ng/ml) and the bull had severe teratozoospermia. The bull remained otherwise healthy and was discharged 35 days after the start of treatment. Eleven months later, the bull was slaughtered because of continued poor semen quality, and the brain, internal organs and testes were examined post mortem.

**Fig. 4:**
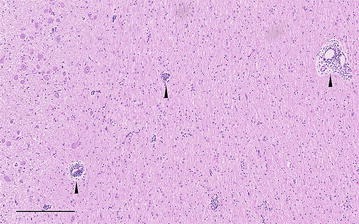
Photomicrograph of the *diencephalon*. Cross section through the *diencephalon* to the left of the midline showing mild non-suppurative perivascular cuffing of small vessels (*arrow heads*). H&E,* bar* 200 µm

**Fig. 5:**
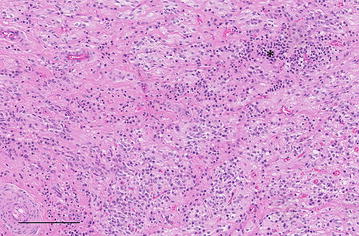
Photomicrograph of the pituitary gland. Longitudinal section through the pituitary gland at the level of the junction between the *pars nervosa* and *pars intermedia*. This junction is vague and there are diffuse lymphocytes and plasma cells. One aggregate is marked with an *asterisk*. H&E,* bar* 100 µm

The pituitary gland weighed 3 g and appeared relatively small but had no other gross abnormalities. The brain, including the hypophysis, the testes, and their accessory glands were fixed in 10% neutral buffered formalin, processed by routine methods for histology, sectioned at 2 µm, and stained with haematoxylin and eosin (H&E). Histologic examination of the *diencephalon* on the left side of the midline showed multifocal perivascular lymphocytic and plasma cell cuffing of small vessels, which was seen predominantly within peripheral white matter tracts of the ventral *diencephalon*. The cuffs formed single-cell to two-cell layers in the Virchow-Robin spaces (Fig. 4). Capillaries in the adjacent grey matter also were frequently associated with small numbers of lymphocytes and plasma cells. There was no neuronal damage. There was mild perivascular cuffing in the ventral white matter of the *diencephalon* to the right of the midline. Occasional extravascular lymphocytes and plasma cells were present in the ventral meninges. Anastomosing thin branches of fibrous tissue were seen between acini in the *pars distalis* of the pituitary gland, and moderate numbers of lymphocytes and plasma cells were present dorsally toward the *pars intermedia*. There was mild to moderate multifocal infiltration of lymphocytes and plasma cells in the *pars intermedia*. The *pars nervosa* and to a lesser extent the infundibulum had multifocal individual and follicular aggregates of lymphocytes and smaller numbers of plasma cells (Fig. 5). Immunohistochemical staining for CD3 (T cells) and CD20 (B cells) (CD3: mouse monoclonal antibody, Dako M7254; CD20: rabbit polyclonal antibody, RB-9013-P, Thermo Fisher Scientific, Reinach) showed a mixed population of stained cells and revealed numerous scattered lymphocytes, which were not clearly evident with H&E staining, in the *pars distalis*. Discrete focal increases in glial cell density with an asymmetric distribution were seen in the *medulla oblongata* on both sides of the midline. The histological diagnosis of mild chronic non-suppurative meningoencephalitis that appeared to be restricted to the ventral *diencephalon* was established. Additionally there was mild to moderate multifocal chronic lymphoplasmacytic hypophysitis with mild multifocal fibrosis.

**Fig. 6:**
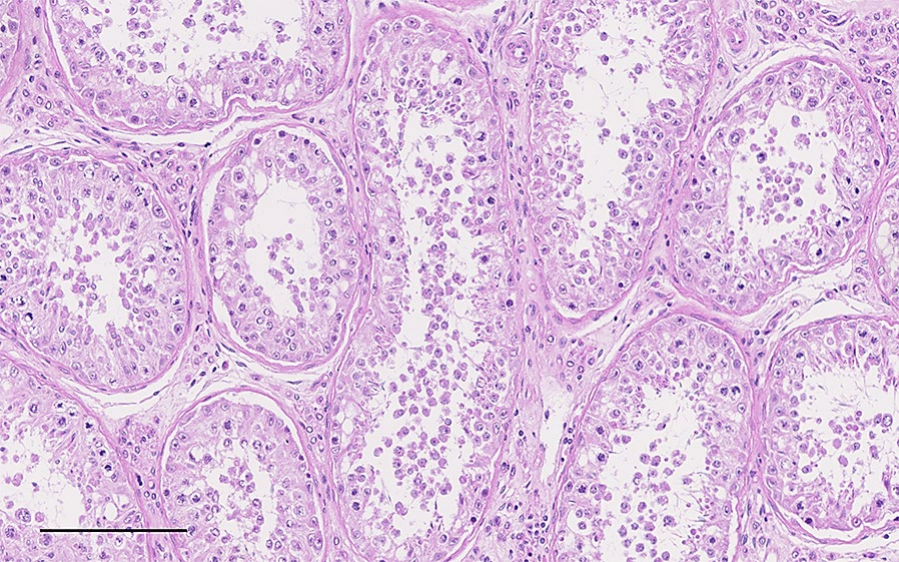
Photomicrograph showing testicular atrophy. Seminiferous tubules of a Holstein–Friesian bull with testicular atrophy attributable to abscess of the hypophysis. The tubules contain exfoliated germ cells and normal spermatozoa are not observed; the majority of cells exhibit spermatogenic arrest prior to the differentiation stage of development (spermiogenesis) H&E,* bar* 100 µm

The testes were symmetrical and each measured 13 × 7 × 4 cm. The left and right testes weighed 296 and 288 g (reference: 267.5 ± 14.4 g [15]), respectively. The seminiferous tubules of both testes contained small numbers of spermatogenic cells of varying and disordered stages of development with occasional multinucleated and degenerated cells suggesting impaired spermatogenesis (Fig. 6). There were no histological signs of orchitis, epididymitis or inflammation of the accessory glands.

The neurological signs described in this case report were typical of those described for cattle with pituitary abscess [1] and pointed to the involvement of several cranial nerves that arise from the *medulla oblongata*. Flaccidity of the tongue was attributable to hypoglossal nerve paralysis, dropped jaw to trigeminal nerve paralysis, dysphagia and salivation to glossopharyngeal and vagal nerve paralysis, head tilt to the right to vestibular nerve paralysis and unilateral ptosis to facial nerve paralysis. Lateralisation of some of the nerve deficits suggested a lesion on the right side of the brain.

The severe bradycardia (32 bpm) was of particular interest. Bradycardia is a common finding in bovine spongiform encephalopathy even though affected cows exhibit signs of extreme nervousness and apprehension [16]. This is thought to be due to pathological changes in the nuclei of the vagus nerve in the *medulla oblongata*, which controls cardiovascular parameters, resulting in vagotonic bradycardia. It is possible that a similar mechanism caused bradycardia in the present case. Lateralisation of clinical signs is typical of listeriosis but also can be caused by abscesses and tumours. Cytological examination of CSF revealed severe purulent inflammation and thus was not indicative of listeriosis, which is characterised by an increase in mononuclear cells in the CSF [17]. CT images showed a focal lesion in the *diencephalon* which, aided by the CSF analysis, was interpreted as an abscess in the ventral *diencephalon*, immediately rostral and dorsal to the hypophysis. To the knowledge of the authors, this is the first CT characterisation of an abscess in the *diencephalon* in cattle. Because of the genetic value of the bull, treatment with amoxicillin, gentamicin and flunixin meglumine was started despite the unfavourable prognosis. This treatment regime has been used successfully at the Department of Farm Animals, University of Zurich, for more than 15 years for the treatment of cattle with listeriosis. The response of the bull to treatment with resolution of clinical signs after 9 days and discharge from the clinic after 35 days was surprising considering that brain abscess in cattle almost always has a poor prognosis. However, it can be assumed that the prognosis depends on a multitude of factors including the size of the abscess, thickness of the abscess wall, type of incriminating bacteria and anatomical location. However, the bull became infertile, which was likely related to decreased luteinizing hormone secretion and testosterone levels caused by inflammatory changes in the *diencephalon*. This was supported by the finding of abnormal spermiogenesis rather than inflammatory changes in the semen. In human medicine, persistent hypophyseal dysfunction also is a problem after treatment of a pituitary abscess [12] and affected patients may require pituitary hormone replacement therapy. The most likely aetiology of the abscess in the *diencephalon* was haematogenous spread of bacteria from bronchopneumonia, which preceded the neurological changes. Bacteria crossing the blood–brain barrier may colonize the CSF. The anatomy of the *rete mirabilis* and its intimate association with the pituitary gland may explain the predilection for pituitary abscesses in cattle [18]. The findings of mild, chronic, non-suppurative meningoencephalitis and mild to moderate hypophysitis do not conflict with the clinical, CSF or CT findings because successful treatment of the bull preceded the post-mortem examination by a year, during which time the bull was clinically healthy.

## Conclusions

This case report stresses the significance of CT in the diagnosis of central nervous system disorders in cattle. The clinical, laboratory and radiographic diagnoses were supplemented by exact anatomical definition of the lesion. However, CT is an expensive technique limited to cases of special interest. This case showed that medical treatment of brain abscess in cattle can be successful.
